# EFFECTS OF PARENTING STYLE IN CHILDHOOD ON MENTAL HEALTH OUTCOMES OF CAREGIVING IN ADULTHOOD: A QUALITATIVE ANALYSIS

**DOI:** 10.1093/geroni/igac059.2548

**Published:** 2022-12-20

**Authors:** Abby Baumbach, M Courtney Hughes, Lily Derain, Yujun Liu

**Affiliations:** Northern Illinois University, DeKalb, Illinois, United States; Northern Illinois University, DeKalb, Illinois, United States; Northern Illinois University, DeKalb, Illinois, United States; Northern Illinois University, Naperville, Illinois, United States

## Abstract

The life course perspective suggests that caregivers of aging parents bring their histories of relationships with aging parents, such as childhood maltreatment, to the care environment. These histories would then impact the dynamics and consequences of caregiving. However, there is a lack of research on the impact of childhood parental interactions on adult relationships with aging parents, particularly in the context of caregiving. The study aims to understand how relationships with caregivers while growing can impact an individual's role as a caregiver later in life. The qualitative study included 47 adult family caregiver survey respondents who care for their parents, with a mean age of 46.7, ranging from 20 to 79 years old. The respondents were asked to reflect on their recent experience of providing care for their loved ones and how experiences with their caregiver growing up may have influenced their current relationship dynamic. The researchers used Dedoose V.9.0.17 to perform a codebook thematic analysis. Themes from the survey analysis linking childhood to current experience included reciprocating good care, performing obligatory care, and stopping the generational transference of negative care. When focusing on specific parental genders when receiving care in childhood, levels of presence and affection stood out as important aspects of their father’s caregiving. Themes associated with maternal caregiving included good relationships, strained relationships, and nurturing. Knowledge about the impact of childhood experiences can help program designers develop interventions to help lessen caregiver burdens that consider childhood care receiving experiences and the challenges and opportunities they present.

